# Acute arthritis of the right temporomandibular joint due to Lyme disease: a case report and literature review

**DOI:** 10.1186/s12903-021-01744-4

**Published:** 2021-08-16

**Authors:** Christina Weise, Matthias C. Schulz, Karin Frank, Marcel Cetindis, Bernd Koos, Hannes Weise

**Affiliations:** 1 Department of Orthodontics, Center of Dentistry, Oral Medicine and Maxillofacial Surgery With Dental School, University Hospital Tübingen, Eberhard Karls Universität, Osianderstr. 2-8, 72076 Tübingen, Germany; 2 Department of Oral and Maxillofacial Surgery, Center of Dentistry, Oral Medicine and Maxillofacial Surgery With Dental School , University Hospital Tübingen, Eberhard Karls Universität, Osianderstr. 2-8, 72076 Tübingen, Germany

**Keywords:** Lyme disease, Temporomandibular joint, Temporomandibular disorder, Differential diagnosis, Lyme borreliosis

## Abstract

**Background:**

Lyme disease is the most frequent tick-borne infectious disease in Europe. It often presents with a wide variety of symptoms. For this reason, affection of the temporomandibular joint (TMJ) caused by Lyme disease (LD) can be misdiagnosed as a common temporomandibular disorder (TMD).

**Case presentation:**

The purpose of this case report of a 25-year-old woman presenting to the Departments of Orthodontics and Oral and Maxillofacial Surgery with extensive symptoms of temporomandibular disorder is to illustrate the delayed diagnosis of Lyme disease which was only made after extensive therapy of the temporomandibular joint. The specialist literature only reports a few cases of patients suffering from Lyme disease with TMJ manifestations.

**Conclusion:**

This case report and the relevant literature review aim to emphasize the importance of accurate request of medical history and differential diagnosis of acute TMJ arthritis and arthralgia. Early interdisciplinary diagnosis of Lyme disease and early antibiotic therapy are essential to avoid misdiagnosis and unnecessary, sometimes invasive, therapies.

## Background

Lyme disease or tick-borne spirochaetosis was first observed in Lyme in Connecticut, USA in 1975. Within a short period of time cases of illness with joint inflammation after tick bites occurred with striking frequency. Skin manifestations of Lyme disease had already been described in Europe by Alfred Buchwald around 1890, but the causal pathogen, Borrelia burgdorferi (Bb), was only officially classified in 1981. It is a bacterial infection caused by the spirochaetes Borrelia species transmitted by the bite of the Ixodes ricinus tick. The bacteria spread lymphogenically and haematogenically in the body manifesting as a multiorgan disease and with different clinical symptoms. Five different species of spirochaetes that can cause Lyme disease are known in Europe. The pathogenic potential of the different Bb species varies [28]. It is the most frequent tick-borne infectious disease. The incidence is increasing in many countries, especially in Europe, North America and countries with a moderate climate. The yearly peak of infections is in July, even at temperatures as low as 1.5 °C [8, 11]. The current number of annual cases of Lyme disease is about 300,000 in the United States and 65,000 persons per year in Europe [1, 22]. The risk of infection is strongly dependent on the weather conditions. Nevertheless, LD is a widespread disease that should be taken seriously [8, 20]. One of the main symptoms of an early manifestation is the erythema migrans (EM) occurring in 89% of the cases. The EM is a bluish-red or red expanding patch, which can be more than five centimeters in diameter, with or without a central punctum. Another clinical manifestation of LD in clinical routine is acute Lyme neuroborreliosis (LN) affecting the nervous system. LN might occur as lymphocytic meningitis, root pain, cranial nerve paralysis (nerve VII) and sometimes as peripheral nerve paralysis [3, 23–25]. In cases with affection of the joints, patients might be diagnosed with Lyme arthritis (LA), which mostly affects the large joints, such as the knee joint causing symptoms like pain and slight swelling [16]. The disease is a result of inflammatory reactions (Lyme borreliosis) and does not produce any toxins. The clinical symptoms of this disease progress through different stages with different manifestations. Symptoms may occur in more than one organ [3, 21, 24]. These authors describe a distinction between early and late manifestation. The early manifestation might appear either localized or disseminated. The early localized stage occurs in 80–90% of the patients and is associated with erythema migrans (EM) [2, 30]. A few days after the infection with borrelia, arthralgia, nausea, fatigue, myalgia, increased body temperature, stiff neck, neurological manifestation and night sweats might be observed. There might be short periods of intense pain in the affected joints, followed by periods of complete recurrence and remission. The early disseminated stage appears after weeks or even months [9]. The heart, nervous system and brain can also be affected. If the orofacial region is involved, the main symptoms are headaches, neurological symptoms like Bell’s paralysis, and/or pain in the masticatory muscles and the temporomandibular joint (TMJ). Facial nerve paralysis is the most frequent cranial neuropathy [23, 27]. Late manifestations are very rare and have a higher risk of a chronic progression.

Laboratory serology is essential for the diagnosis of Lyme disease. Serological diagnostics should only be requested if there is sufficient clinical suspicion. For serological examinations serum antibody detection methods such as enzyme-linked immunoassay (ELISA) and immunofluorescent assay (IFA titer) are used. Serological detection is difficult because the diagnostic methods are not standardized and have a low sensitivity. Thus, it is often a tentative diagnosis. Furthermore, there is an inability to detect antibodies in the early stages of LD meaning that an infection might be present despite a negative test result [14]. Furthermore, serological evidence can no longer be provided after antibiotic therapy. In the case of an infection with BB, no specific degenerative and inflammatory evidence is found in the affected joint, neither radiographically nor histopathological [21]. Antibiotic treatment should be administered over a period of 14 days [20]. According to the guidelines, doxycycline is as effective as beta-lactam antibiotics in early neuroborreliosis regarding regression of neurological symptoms with the same tolerability. There are no valid evaluable studies on the effectiveness of combination treatments of antibiotics [20]. The most common non-dental pain arises from temporomandibular disorders (TMDs), having become widespread disorders. TMD has similar symptoms to LD, such as undifferentiated neuralgic facial pain, limited mouth opening, disorder of mandibular mobility, ear pain, joint sound and sometimes hypermobility of the joint (Table 1) [7]. Persistent pain can lead to a reduction in health-related quality of life and to psychosomatic disorders. Therefore, patients suffering from LD can be misdiagnosed with TMD and consequently undergo inappropriate treatment. If the LD persists for a long time, it can lead to irreversible chronic manifestations [14]. Heir and Fein [11] showed that in rare cases LD has a manifestation at the temporomandibular joint (TMJ). They emphasized that patients with LD should undergo special treatment and some form of specific recording of medical history such as a questionnaire [11]. In LD the TMJ is the fourth most frequently affected joint [26]. So far, there is a paucity of information in the literature and the relationship between TMD and LD is still unclear. A new case with these symptoms in serologically confirmed LD is reported below. This is intended to increase the awareness of dentists to patients who present with TMD symptoms in combination with evidence of LD, so that they can be referred to an appropriate specialist.

**Table 1:** Vascular and non-vascular intracranial cause of orofacial pain

*Vascular and nonvascular intracranial cause of orofacial pain*
Headache associated with vascular intracranial disorders (IHS/ICHD-3 code 6.1 to 6.6)
Headache associated with nonvascular intracranial disorders (IHS/ICHD-3 code 7.1 to 7.8)
*Primary headache disorders*
Migraine (IHS/ICHD-3 code 1.1 to 1.6)
Tension-type headache (IHS/ICHD-3 code 2.1 to 2.4)
Cluster headache and other trigeminal autonomic cephalalgias (IHS/ICHD-3 code 3.1 to 3.5)
*Neuropathic pain*
Episodic neuropathic pain (IHS/ICHD-3 code 13.1.1, 13.2, 13.3, 13.9)
Continuous neuropathic pain (IHS/ICHD-3 code 13.1.2, 13.10, 13.11, 13.12.2)
Dysesthesia
*Intraoral pain disorders*
Odontogenic pain
Non odontogenic pain
Oral mucosal pain
*Temporomandibular disorders*
Temporomandibular joint disorders
Masticatory muscle disorders
*Extracranial causes of orofacial pain and headaches*
Pain stemming from tissues or organs in the head and neck (IHS/ICHD-3 code 11.1, 11.3 to 11.5)
Pain stemming from systemic disease (IHS/ICHD-3 code 13.12.1)
*Cervicogenic mechanisms of orofacial pain and headaches*
Common cervical spine disorders (IHS/ICHD-3 code 11.2, 11.8, 13.2, 13.4)

Classification structure of orofacial pain conditions from the 5th edition of the American Academy of Orofacial Pain guidelines [13]; Abbreviations: IHS: International Headache Society; ICHD-3: International Classification of Headache Disorders

## Case presentation

**Fig. 1:**
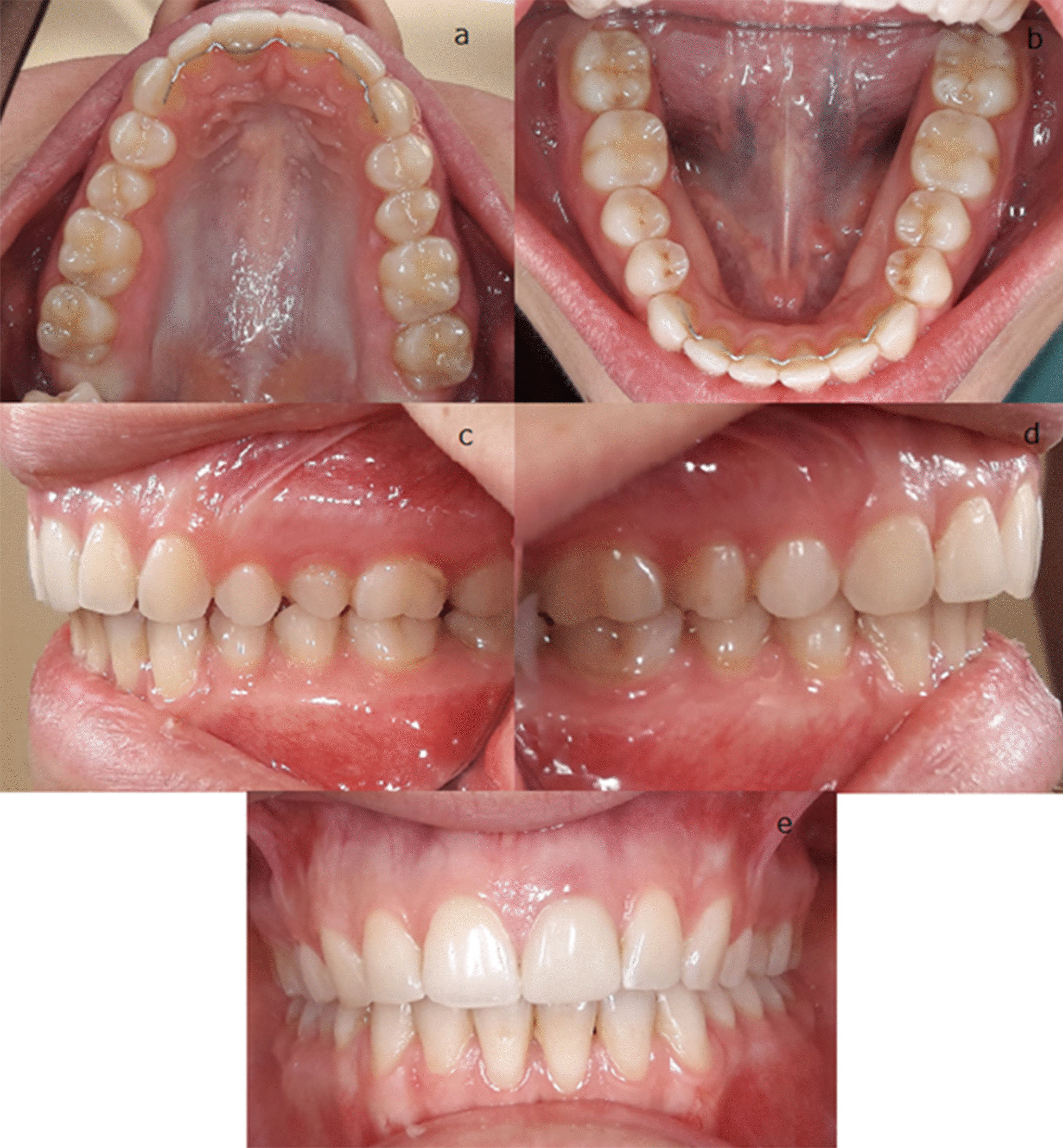
Intra-oral photographic state: **a** Upper jaw occlusal; **b** Lower jaw occlusal; **c** left side occlusion **d** right side occlusion **e** frontal occlusion

**Fig. 2:**
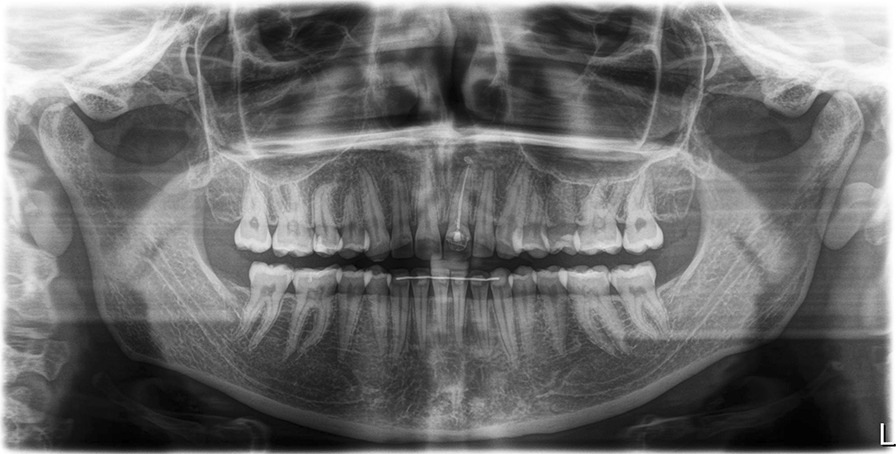
Panoramic radiograph of the patient showing symmetric but flattened condyles with a slightly widened TMJ gap on both sides

This case report describes a 25-year-old female patient presenting to the Department of Orthodontics at the Centre of Dentistry, Oral and Maxillofacial Surgery at the University Hospital Tübingen due to acute pain in the right TMJ and mouth opening disorders in 2019. There were no general diseases or allergies in her medical history. The patient stated that she did not consume alcohol or nicotine; she was taking contraceptives. Over a period of 5 years the patient’s distal bite was treated with removable and fixed orthodontic appliances. The initial examination showed fixed retainers in both the upper and lower jaws (Fig. 1a + b). Despite orthodontic treatment, there was still a slight asymmetric distal occlusion on both sides (Fig. 1c + d). This was slightly more pronounced on the left due to slight mandibular tilt to the left (Fig. 1e). The mouth opening disorder severely restricted the patient in her everyday life. Previously, craniomandibular dysfunction was assumed due to multiple grinding facets on the teeth and bruxism. Therefore, a bruxism splint was already worn at night. The initial panoramic radiograph is shown in Fig. 2.

Three months later, the patient reported that she woke up with severe pain in her right TMJ after sleeping and was not able to close her mouth. When repositioning the lower jaw by herself a very loud cracking sound at the right TMJ occurred. After that, she had a mouth opening disorder and constant pain. The patient gave this constant pain a score of 7 on the verbal numeric rating scale (VNRS) (0–10). She was prescribed muscle relaxant therapy with methocarbamol twice a day. Although the pharmaceutical treatment with methocarbamol resulted in some slight pain relief, it also had some general medical side effects such as circulatory weakness and dizziness. The patient was also referred to the Department of Orthodontics. Due to severe pain a functional analysis of the TMJ was not possible. The mouth opening was limited to 20 mm active and 30 mm passive causing extreme pain. Thus, a muscle spasm was suspected. A dental splint combined with intensive physiotherapy and mouth-opening exercises continued to be recommended as therapy. Mouth-opening exercises and physiotherapy led to a clear deterioration of the condition.

**Fig. 3:**
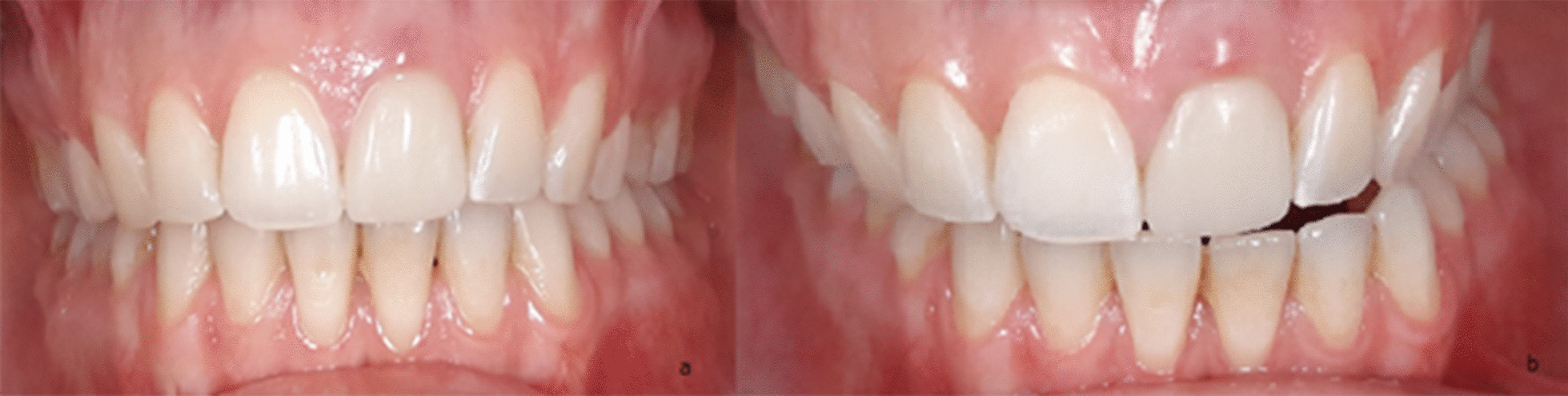
Intraoral picture **a** centric occlusion; **b** deviation of the lower jaw to the left

Three months after the first symptoms, the patient showed an acute deterioration of her health status. The main symptom was ever-increasing pain in the right TMJ, which worsened throughout the day. She rated the pain 8 on the VNRS. She also showed an increasing active mouth opening restriction to 20 mm and a habitual deviation of the lower jaw to the left (Fig. 3).

**Fig. 4:**
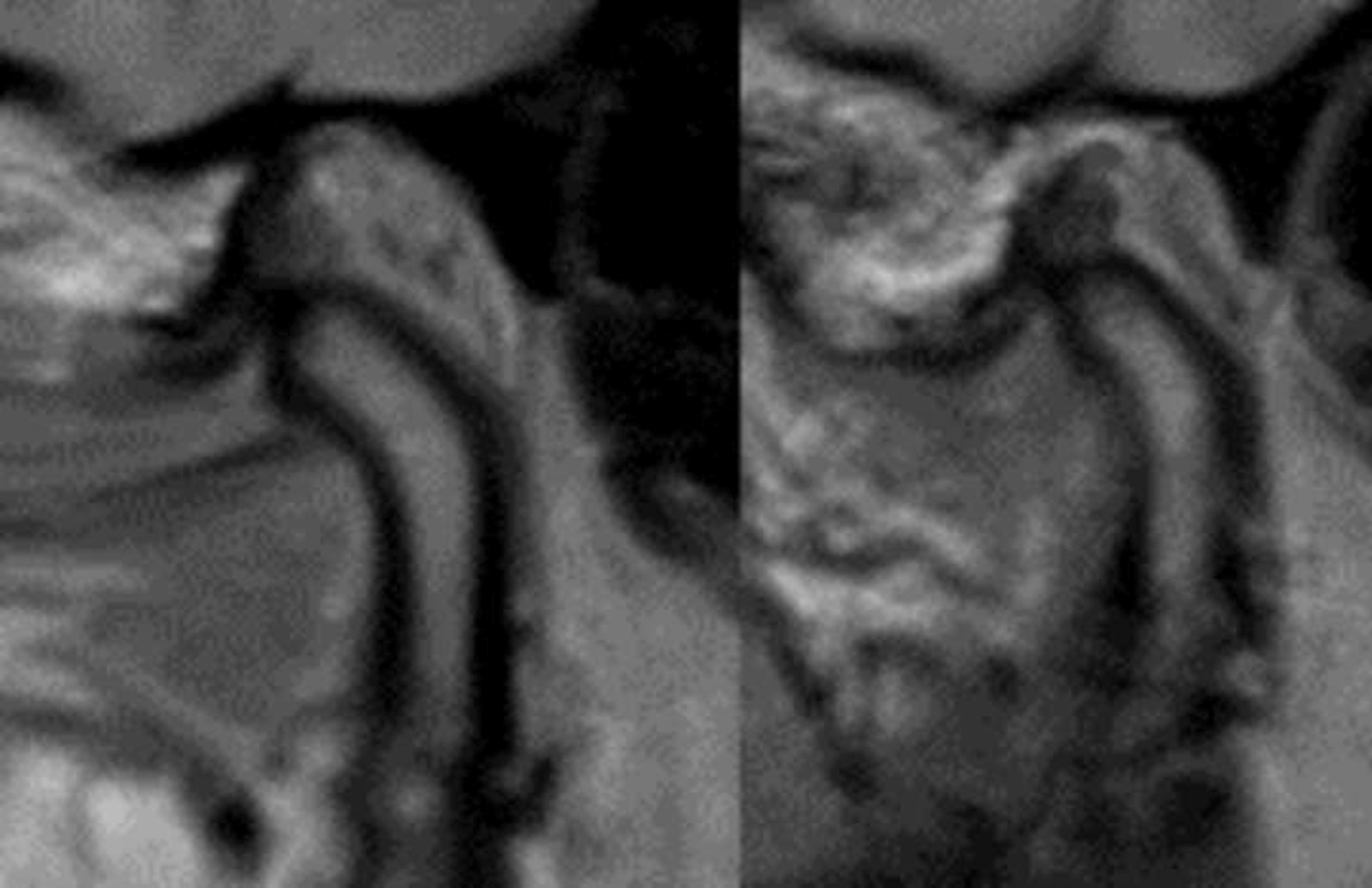
MRI open mouth left (12/19)

**Fig. 5:**
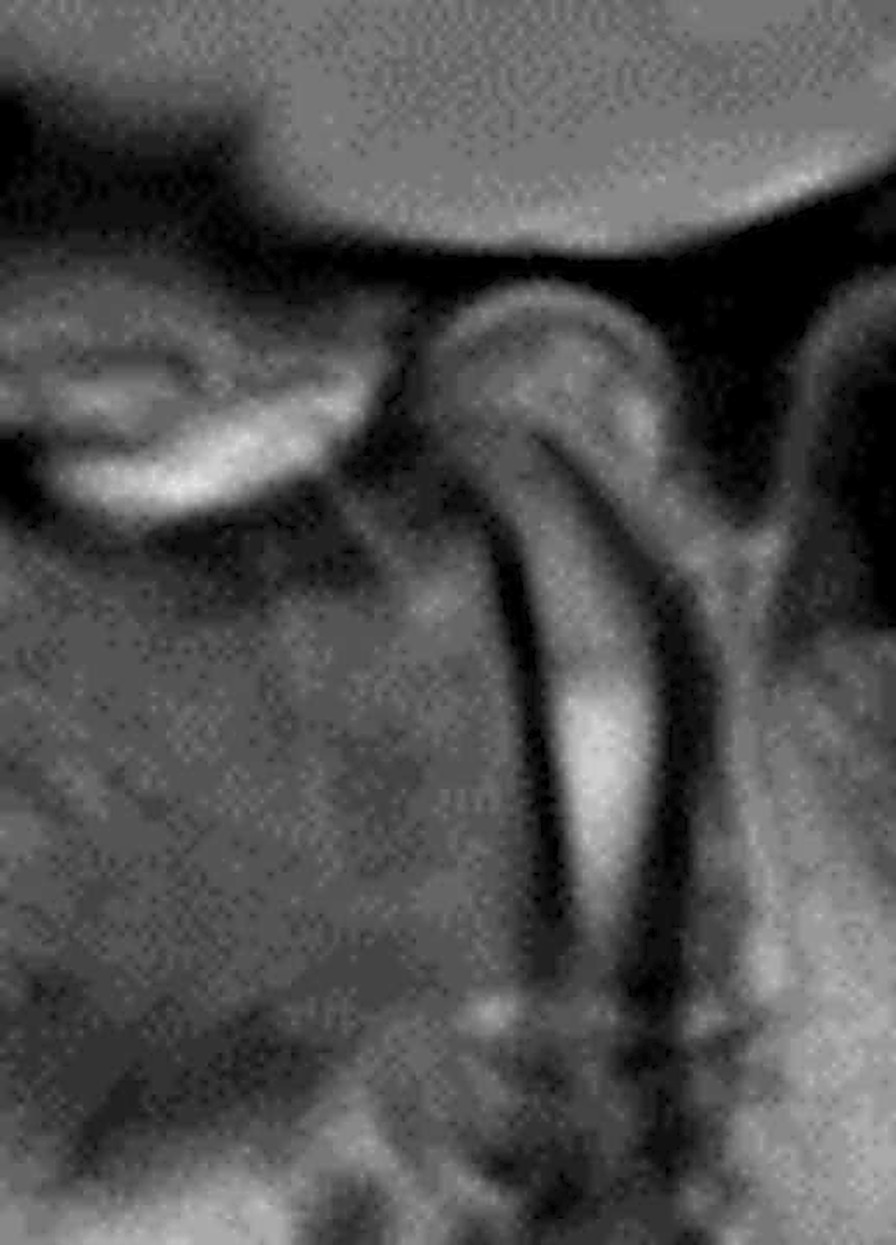
MRI open mouth right (12/19)

**Fig. 6:**
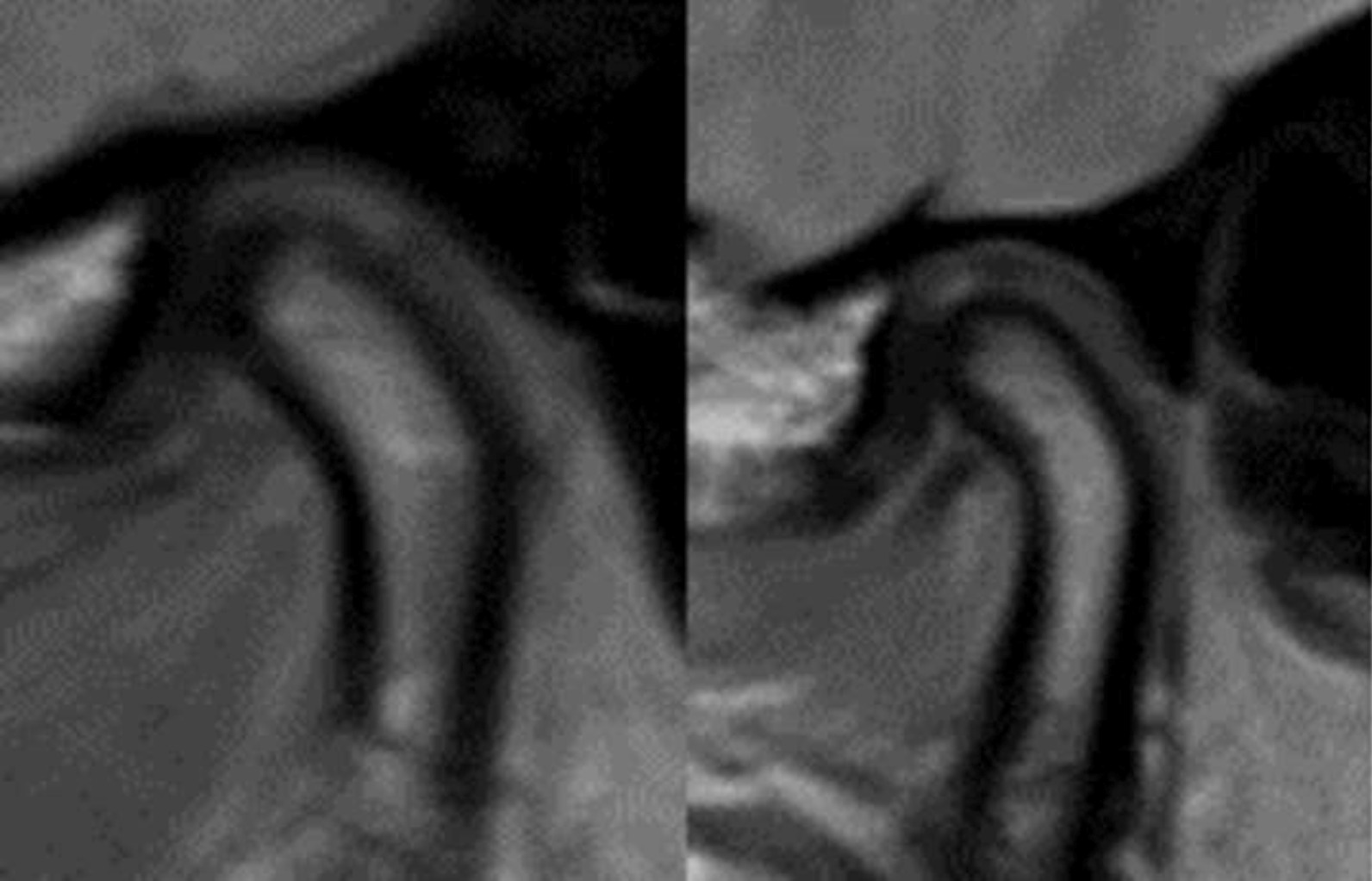
MRI closed mouth left (12/19)

**Fig. 7:**
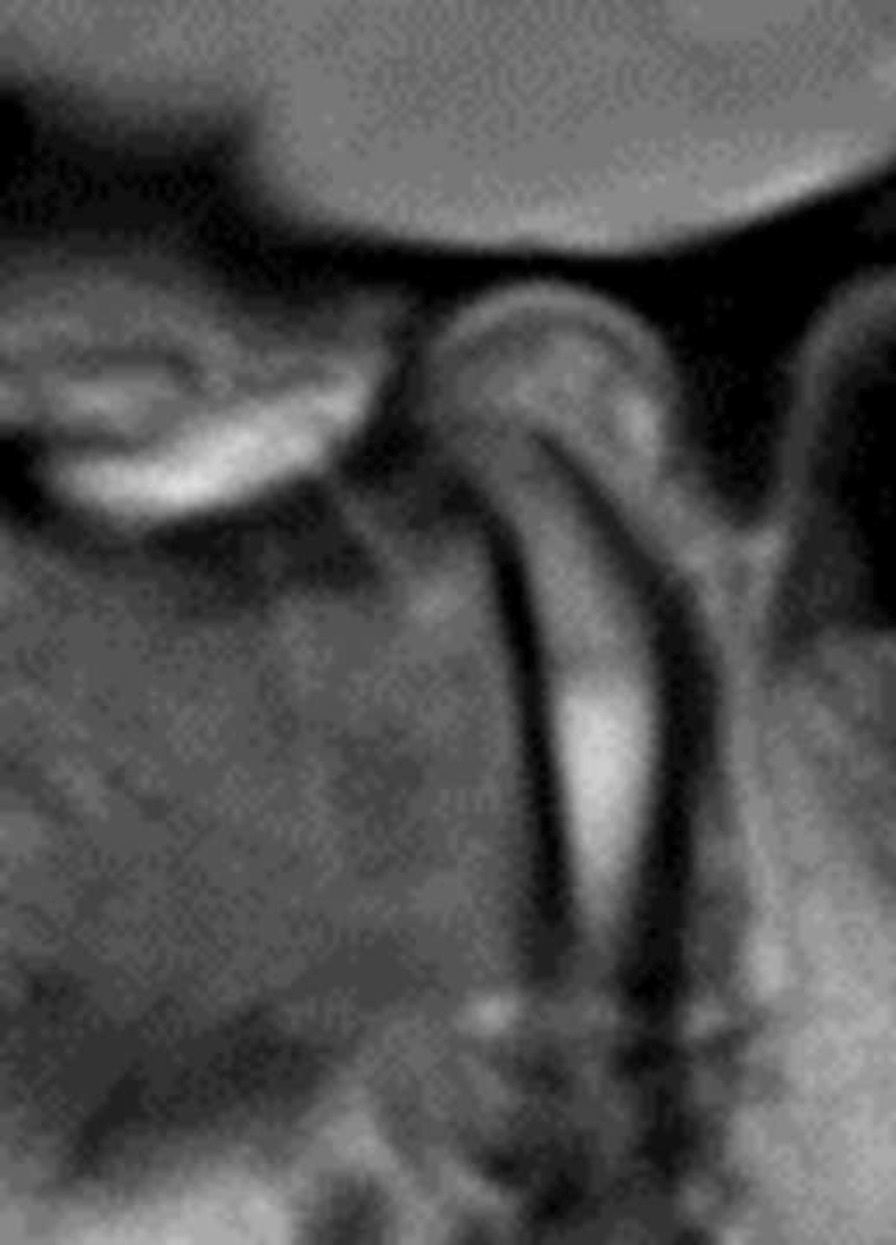
MRI closed mouth right (12/19)

The functional and occlusal analysis of the stomatognathic system showed strong dorsal load vectors of the TMJ on the right side. The ventral and caudal traction of the joint was consistently associated with severe pain on the right. Lateral movements of the lower jaw were restricted and painful. Protrusion movement was not possible. There was neither crepitus nor cracking of either TMJ. The patient had a persistent pain-relieving abduction and outward rotation of the left lower mandible (Bonnet position) (Fig. 3b). The suspected diagnosis at this time was a total ventral deviation of the discus on the right side without reduction and with active mouth opening that was painfully blocked. There was a static contact of the first premolars with the second molars on both sides. The dynamic occlusion had canine guidance on the right side and guidance over the first premolar on the left. The bite position showed an Angle Class II left 3/4 premolar width (PW) right and ¼ PW left with an overjet of 4 mm and an overbite of 2 mm. Further radiological diagnosis was performed using an MRI scan (Figs. 4, 5, 6, 7).

The radiology findings showed a discrete erosion of the right mandibular condyle and a bone marrow oedema of the right mandible with increased contrast uptake as well as minor joint effusion in the right temporomandibular joint. Furthermore, an anterior dislocation of the right articular disc with suspicion of a tear in the ligament with closed and open mouth was identified. In the dynamic sequences, there was slightly restricted translational forward movement with mouth opening on the right side. The position of the left TMJ with open and closed mouth was unremarkable. Thus, the findings of the MRI confirmed osteoarthritic changes of the mandibular condyle and joint effusion on the right side corresponding with constant pain. The treatment goals were to prevent the progression of structural damage to the right temporomandibular joint, to improve the mouth opening and to reduce the lateral deviation. Therefore, the patient was prescribed NSAID (Ibuprofen 400 mg) 3x/d to reduce the pain, a bite splint in the lower jaw for 24 h per day, soft diet and regular adjustment of the splint approximately every 2 weeks.

Despite these therapies the patient complained of increasing pain in the right TMJ and a worsening of her general condition. The patient would then stop the splint therapy at her own discretion. She was also referred to the Department of Oral and Maxillofacial Surgery of the University Hospital Tübingen. The pharmaceutical treatment with NSAID 3x/d was changed to Diclofenac 50 mg 2x/day. The new medication did not achieve pain relief and, unfortunately, the patient suffered an allergic reaction to Diclofenac and resulting in an exanthema, swelling of the oral mucosa and nausea.

**Fig. 8:**
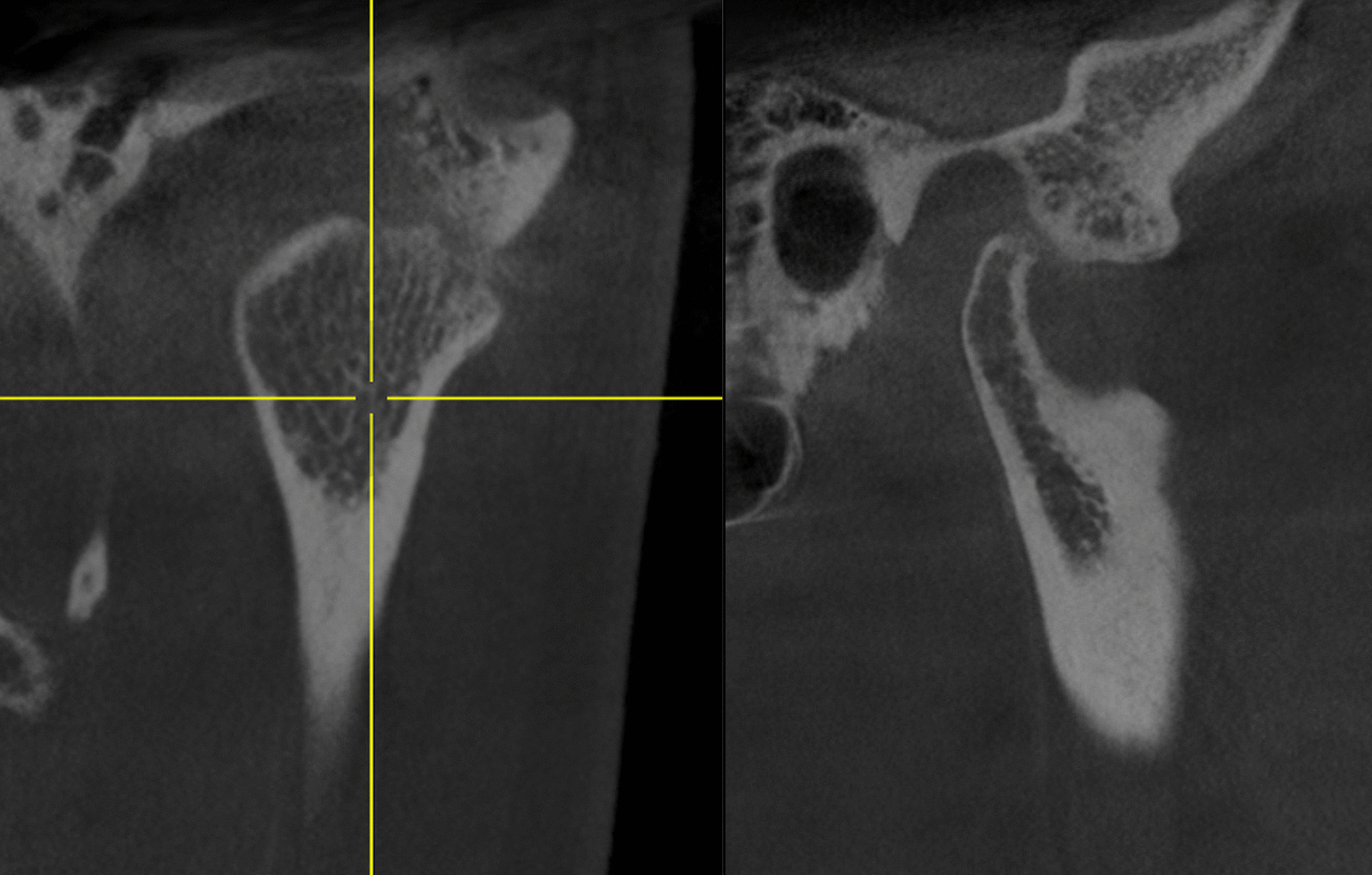
CBCT right TMJ (01/20)

**Fig. 9:**
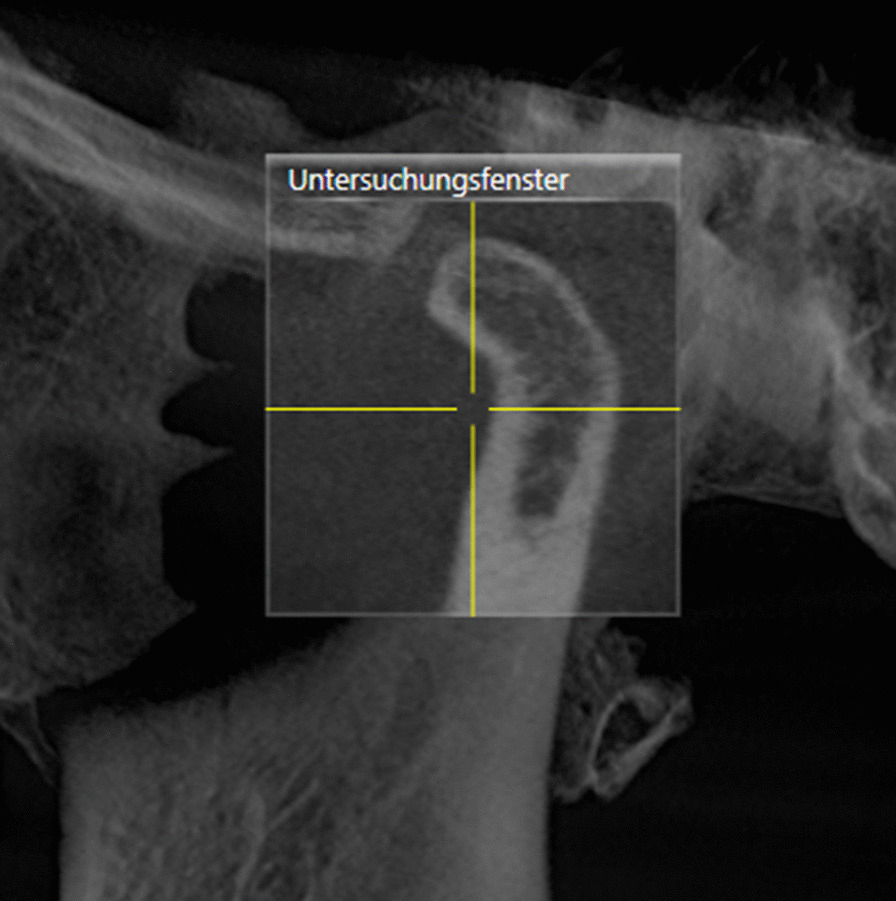
CBCT left TMJ (01/20)

For further diagnostic investigation, a cone beam computed tomography (CBCT) was performed and showed bone erosion of the right condyle of the TMJ. The image revealed an arthropathic condylar destruction on the right seen as a pointed structure; the cortex is only partly visible. The left mandibular condyle did not show pathologic findings (Figs. 8 and 9).

**Fig. 10:**
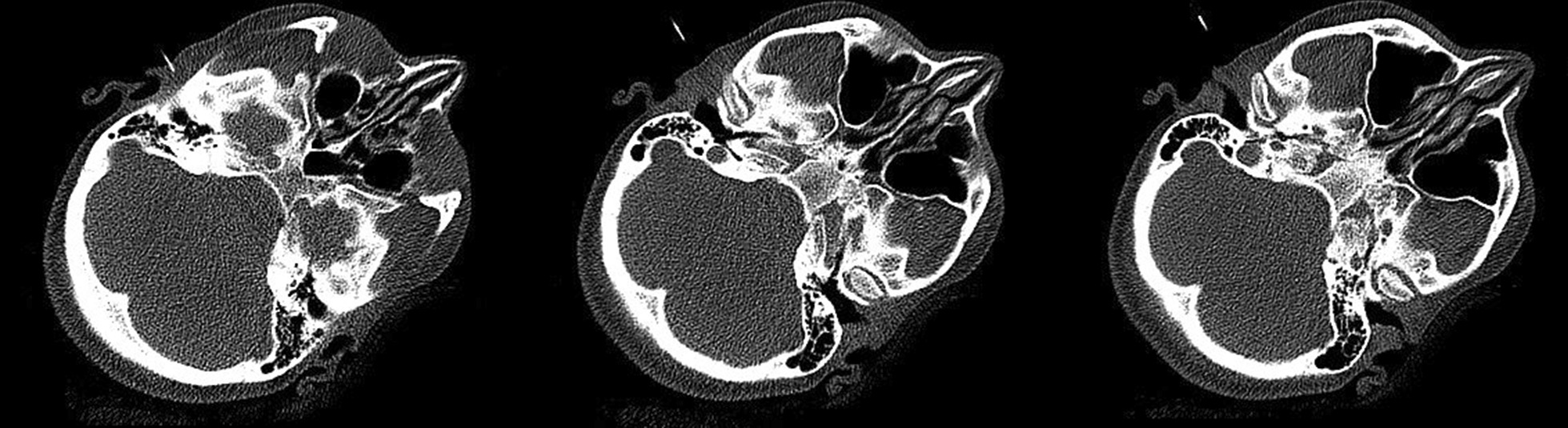
CT scan with cortisone infiltration (01/20)

Based on the radiological findings a mandibulo-maxillary fixation (MMF) with elastic loops was applied to stabilize the occlusion. The patient was instructed to follow a liquid diet and intensive physiotherapy was prescribed. However, her condition failed to improve. The MMF was then removed and the jaws were fixed using wire ligatures. This fixation and immobilization of the mandible led to a remarkable improvement for the first time in the period of treatment. For further clinical diagnostics the patient was referred to the Department of Rheumatology of the University Hospital Tübingen. The rheumatoid serology did not yield any findings indicating a rheumatological disorder. The laboratory examination showed a slightly increased CRP-value of 0.60 mg/L. The HLA-B27 examination was negative. The patient's serologic analysis showed a low estrogen level and thus, the contraceptives were discontinued. Further therapy was discussed by an interdisciplinary board (Rheumatology, CMF-Surgeons and Orthodontics) that proceeded to recommend lavage or cortisone injection into the affected TMJ. A cortisone injection in the right TMJ was performed shortly after the interdisciplinary discussion (Fig. 10). After cortisone injection, the patient showed a significant improvement generally and the active mouth opening increased to up to 23 mm. However, pain at maximal mouth opening was still felt in the right TMJ.

Management recommendations continued to include a soft diet in order to relieve the TMJ, no forced mouth opening exercises, physiotherapy and an attempt without MMF. Three months later, a blood test revealed a positive incidental serology finding for Bb IgG and IgM antibodies. Based on the serological finding, the diagnosis of Lyme arthritis affecting the right TMJ was confirmed. Cephalosporins (Cefuroxime 1.5 g 3x/d) i.v. for at least 3 weeks was the immediate treatment of choice. Interestingly, the patient could not recall any tick bite. After one week of antibiotic therapy, the patient stated a clear improvement of her condition. A further functional examination of the temporomandibular joints after antibiotic therapy revealed a not reproducible, persistent cracking sound on the right side during active mouth opening.

**Fig. 11:**
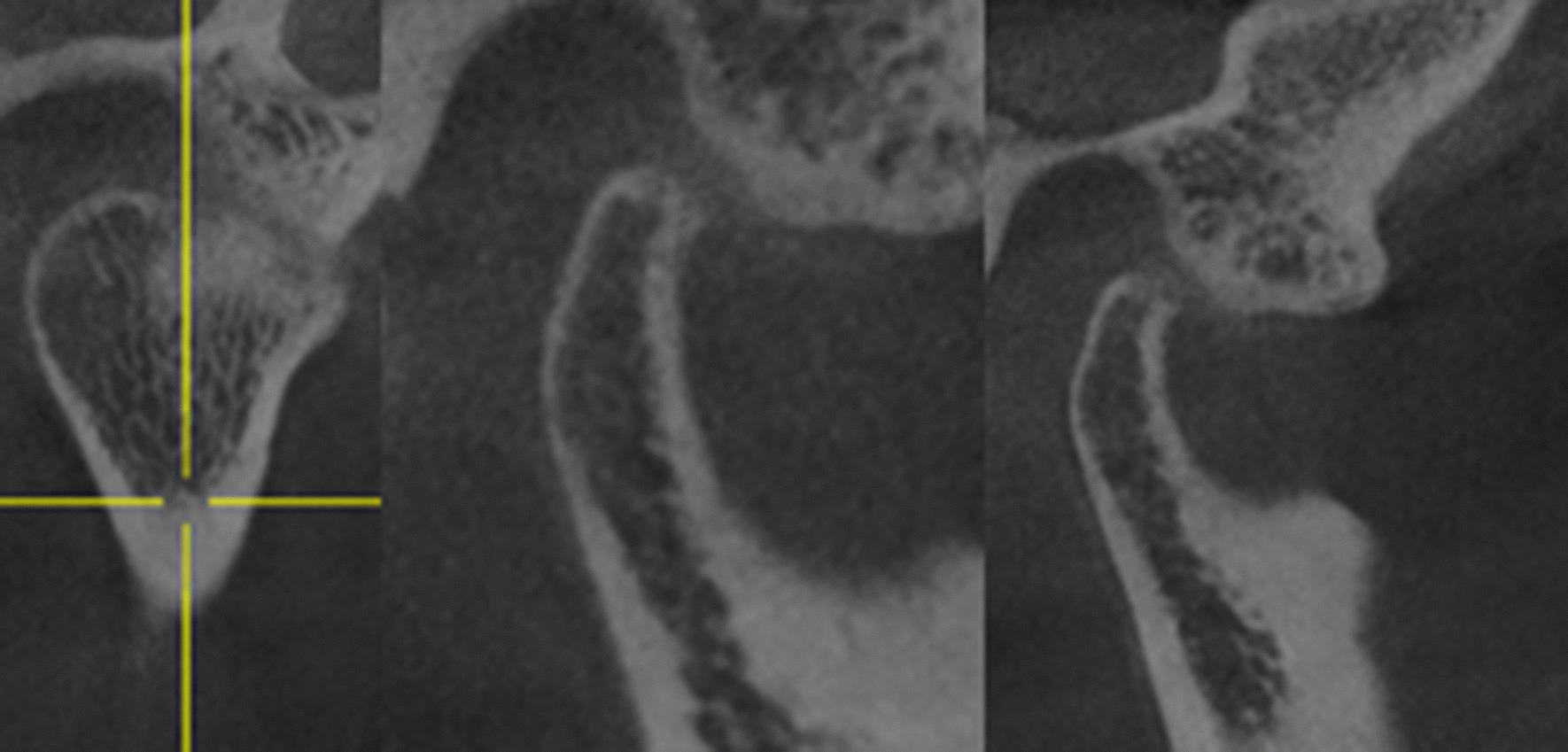
CBCT right TMJ (07/20)

A CBCT scan obtained three months after the antibiotic therapy confirmed a pronounced improvement of the situation (Fig. 11). The image of the right temporomandibular joint showed an almost continuous cortex with only a few residual lesions. Thus, the condyle had significantly improved compared to the previous CBCT scan seven months earlier. The functional and occlusal analysis showed that the lateral restraint no longer existed. However, the static contact and the dynamic occlusion on both sides was unchanged compared to the situation seven months ago. Furthermore, the bite position displayed an Angle Class II left 1/4 PW right and ¾ PW left with an overjet of 4 mm and an overbite of 2 mm. The patient still reported a cracking sound during mouth opening on the right side only but this was not reproducible during the clinical examination.

## Discussion and conclusions

Until today, the manifestation of LD in the TMJ has not been extensively described in the literature (Table 2). Harris [9] was the first author to described symptoms in the TMJ arising in combination with LD in a younger patient. In the United States, only 5 authors have reported the manifestation of Lyme disease in the temporomandibular joint since 1988 [9, 11, 14, 15, 32]. In Germany, only two other case reports have been presented. Lešničar and Žerdoner [16] presented two cases of LD affecting the TMJ that occurred between 2000 and 2003 for the first time in Slovenia. Both patients showed fatigue, muscle pain and arthralgia in the TMJ region. The therapy was intravenous ceftriaxone after detection of the acute Bb infection by positive serum markers. The authors suggested that in Slovenia joint involvement is present in 15% of the cases. This statement is consistent with reports of joint involvement in LD across Europe [6]. However, it can be stated that the occurrence of these disorders is much more frequent in the United States, whereby joint involvement appears to be a more frequent clinical sign of LD in the USA than in Europe [16]. Almost all cases showed a restriction in mouth opening and an improvement of the symptoms after antibiotic therapy alone.

**Table 2:** Review of the available literature

Authors	Patient	Region	Symptoms	Diagnostic/therapy
Burkhard et al. [4]	31, f	Germany	Pain left TMJ radiating into the upper and lower teeth Difficulty with mouth opening (32 mm) Sensation swelling left side of face Redness left cheek to chin Bruxism Fatigue	MRI: Idiopathic facial nerve palsy with inflammatory changes in the parotid and submandibular glands → Zovirax (5 × 800 mg/7 d) ELISA serology positive IgM and IgG
Xie et al. [32]	16, m	United States	Left temporomandibular joint, right patella pain Dislocation, bilateral forearm pain Fatigue Left-sided Bell’s palsy Low grade fever Left mandibular pain	Western blot: positive Lyme antibodies Doxycycline Bilateral TMJ diagnostic arthroscopy followed by arthrocentesis with joint lavage and intra-articular (IAC) corticosteroid injections of the TMJ’s
te Veldhuis et al. [27]	61, f	Netherlands	Facial pain Restriction of mouth opening to 38 mm Impaired speaking ability Inability to close lips Bilateral peripheral facial nerve paresis Weakness of left arm	Laboratory testing: positive IgG and IgM antibodies 3-weeks intravenous ceftriaxone; 2 g per day
Wolanska-Klimkiewicz et al. [31]	50, f	Poland	Tick bite was noticed Swelling of eyelids (upper and lower), cheek, neck after 12 h ? weeks after the bite, the condition worsened: Pain in joints, muscles, conjunctivitis, hypersensitivity to light, dental symptoms, pain in the chest and heart area with increased blood pressure 5.5 months after tick bite: erythema on lower limb Neurologic symptoms: paralysis of the left side face, numbness in the right neck	Antihistamine drugs Compresses with aluminum acetotartrate (Altacet) Synthetic corticosteroid (Elocom) Oral heart supplements Macrolide antibiotic ELISA test: positive IgM and antibodies Doxycycline (Unidox), Metronidazol Amoxicillin (Amoksiklav)
Lee et al. [15]	8, m	United States	TMJ pain Mouth opening difficulty (max. 10 mm) Stiff neck Frequent headache Bilateral knee joint swelling	400 mg Ibuprofen every 6 h Warm compresses CT Scan: inflammatory sites in both TMJs Western blot: High serum antibody: Amoxicillin for 21 days (50 mg/kg)
Lesnicar and Zerdoner [16]	59, f	Slovenia	Erythema migrans Fatigue Pain in shoulder, knee, hip joints Pain in right TMJ Difficulty with mouth opening (max. 20 mm) Fever Uneven surface of mandibular head TMJ Swollen joints	ELISA serology positive IgM and IgG Ceftriaxone for 3 weeks
	52, m	Slovenia	Fatigue Myalgia in the extremities Pain in both TMJs Pain in left arm	ELISA, Western blot Serological positive Bb IgM and IgG antibodies Ceftriaxone daily dosage of 2 g for 3 weeks
Vesper et al. [29]	49, f	Germany	Pain left TMJ Fever Swollen left TMJ Difficulty with mouth opening (max. 20 mm) Non-occlusion left side Bonnet-protective posture Left-sided TMJ arthritis without an indication of degenerative disease	Rocephin parenteral 2 g/d Infection with Bb through tick bite was known → Diagnosis was only confirmed 5 years after tick bite by serological detection Doxycycline for 7 d (twice in a period of 3 months)
Heir and Fein [11]	49, f	United States	14 months of neurological symptomology Facial pain Facial numbness Left Eye pain Numbness of extremities Joint pain Concentration problems	Oral amoxicillin, 2 years after the beginning of appearance of symptoms Herxheimer reaction after antibiotic therapy Inpatient admission to hospital: Intravenous ceftriaxone sodium
	28, f	United States	Facial pain Pain in the left TMJ Migraine headache Neck pain Visual problems Upper body numbness	Antibiotic therapy
	34, f	United States	Pain of chin + forehead Pain of right cheek Paralysis of the right lower side of face	
Lader [14]	33, w	United States	Headache Paresthesia in posterior cervical region, hands Stiffness and pain in neck Pain in right TMJ + intermittent click Pain masticatory muscles	Chiropractor: treatment failed 12 medications (e.g. Naproxen (Anaprox, Ibuprofen, Methocarbamol) Dentist: Splint therapy → failed Serology Test: first was Bb negative: Penicillin therapy, lidocaine injection → second test positive
Harris [9]	35, f	United States	Pain in right jaw Right lateral pterygoid muscle slightly tender to palpation Difficulty with mouth opening (max. 25 mm) Recurrent episodes of arthralgia in shoulders, elbows, wrists Red spots on chest Positive LD diagnosis: wandering arthritis, erythema chronicum migrans	Hot compresses Soft food diet Medication: muscle relaxants Bilateral tomograms of TMJ: normal left joint, irregularities with reactive sclerosis of articular surface right condylar Tetracycline 500 mg for 2 weeks ELISA serology positive IgM and IgG

Review of literature of case reports who describe Lyme Disease with temporo-mandibular joint manifestation; Abbreviations: m = male; f = female

Often tick bites are not noticed by the affected patients or they cannot remember a bite in the past. Consequently, LD with affection of the TMJ very often is misinterpreted as a TMD. In the case presented here, the tick bite was unnoticed and the symptoms were interpreted as those of a TMD. The warning symptom EM was absent. However, the possibility of missing symptoms should be considered in every patient and should not be a criterion for excluding a LD diagnosis. Therefore, the presented case aims to raise the awareness of this issue. Lader [14] described a case in which Lyme disease was wrongly diagnosed as a temporomandibular joint disorder. Osiewicz et al. [19] discovered that there is a high prevalence of TMD symptoms in patients with LD. The risk of this disease is that it sometimes only becomes apparent in its late phase [17, 25].

Symptomatically, the patient presented with acute temporomandibular joint arthritis in the classical sense without an affection of any other joint. As the patient did not improve with conventional therapy, a variety of differential diagnoses and causes had to be discussed with an interdisciplinary board. The patient was not aware of any tick bite, nor had a Lyme disease been diagnosed before the onset of the acute temporomandibular joint arthritis. It can be assumed that there must have been an infection with borrelia in the right temporomandibular joint for a longer period of time before the symptoms occurred. This circumstance has been described before [11, 16, 31]. It can be assumed that the overloading of the TMJ due to bruxism exacerbated and intensified the manifestation at the TMJ. Osiewicz et al. [18] described a connection between the influences of bruxism in predicting the presence of TMD in patients with LD.

Cortisone injection in the right TMJ led to a significant pain relief and an improvement in the general condition of the patient, whereas splint therapy did not cause any amelioration of symptoms. It can be suggested that cortisone therapy alleviated the inflammation in the TMJ but did not eliminate the cause of the complaints. The initiation of antibiotic therapy ultimately led to a long-term improvement, which could be objectified by the CBCT, which showed the recovery of the right mandibular condyle. Xie et al. described a more invasive therapy in LD patients with affection of the TMJ. They recommended a combined approach comprising antibiotic therapy with doxycycline i.v., arthroscopy of the affected joint followed by arthrocentesis with joint lavage and intra-articular corticosteroid injections in combination with pain medication [32].

This case has confirmed that an early antibiotic treatment is important and prevents recurrent arthralgia. This symptom might be caused by Bb in the synovia of the infected joint. A Ceftriaxone-based parenteral antibiotic therapy per day for three weeks is recommended [5]. In order to investigate whether the patient has a chronic LD activity or whether this is a consequence of the infection, clinical and laboratory examinations would have to be carried out again after a certain period of time following diagnosis. Furthermore, it is important to clarify whether there are long-term arthritic changes in the TMJ. The risk of chronic LD is recurrence and poor therapeutic response. te Veldhuis et al. [27] recommended physical therapy to reduce the orofacial pain and limited mouth opening as well to supplement antibiotic therapy.

Heir and Fein [11] described three cases and the possibility of a Jarisch–Herxheimer reaction associated with antibiotic therapy in dental procedures. The Jarisch–Herxheimer reaction is a reaction of endotoxine like products released by the death of bacteria or spirochetes during antibiotic treatment. Symptoms are fever, chills, rigor, hypotension headache tachycardia, hyperventilation, vasodilation with flushing, myalgia and exacerbation of skin lesions. These clinical symptoms are often misinterpreted as an allergic reaction to the antibiotic therapy. They also highlight the fact that Bb can remain inactive and unknown in the tissue for a long period, ultimately emerging as a Jarisch-Herxheimer reaction. Furthermore, the Bb has an affinity to connective tissue such as the periodontium. There is a risk of surgical intervention involving the periodontium. A surgical procedure or a root canal treatment might cause delayed healing because scarring can increase symptoms [10–12].

The literature highlights antibiotic therapy as the best therapy available for the treatment of LD [11]. So far, no exact therapy for TMJ involvement in LD has been reported in the literature. The prerequisite for a confirmed diagnosis is a well-founded diagnosis, a structured medical history and findings with accompanying diagnostic therapy. Lyme disease-associated orofacial pain poses special challenges for an interdisciplinary team as well as for the patient due to the variety of possibilities of differential diagnoses. Furthermore, the treatment of TMJ problems always focuses on interdisciplinary treatment and an individual therapy concept for each patient. It is important to think "outside the box" and think ahead, especially when making a diagnosis. In the case of unclear TMJ problems and when the TMD treatment is not successful, the possibility of a LD infection should definitely be considered as a differential diagnosis. It is important to bear in mind that the current number of annual cases of Lyme disease is about 300,000 in the United States and 65,000 persons per year in Europe [1, 22]. This means there is a high prevalence of LD and, consequently, possible affection of the TMJ. If the patient is referred to a specialist in time by a thoughtful practitioner, delay in diagnosis and unnecessary treatments could be avoided.

## Data Availability

The original data is available from Dr. Christina Weise (christina.weise@med.uni-tuebingen.de) on reasonable request. All data and material is accessible on a local server of the Departments of Orthodontics and the Department of Oral and Maxillofacial Surgery at the University Hospital Tübingen, Germany.

## Abbreviations

Bb: Borrelia burgdorferi
CBCT: Cone Beam Computed Tomography
CRP: C-reactive protein
EM: Erythema migrans
LA: Lyme arthritis
LD: Lyme disease
LN: Lyme neuroborreliosis
MRI: Magnet Resonance Imaging
NSAID: Nonsteroidal anti-inflammatory drugs
Pw: Premolar width
TMDs: Temporomandibular Disorders
TMJ: Temporomandibular joint
VNRS: The verbal numeric rating scale
